# Perceptions of oral corticosteroid use for children with asthma in a survey of US caregivers

**DOI:** 10.3389/fped.2025.1608425

**Published:** 2025-10-07

**Authors:** Nina C. Ramirez, Vivian Hernandez-Trujillo, Kaharu Sumino, Dennis Williams, Mitu Patel, Donna D. Gardner

**Affiliations:** ^1^ Department of Medical Education, Nova Southeastern University Dr. Kiran C. Patel College of Osteopathic Medicine, Fort Lauderdale, FL, United States; ^2^ Asthma and Allergy Associates of Florida, Pembroke Pines, FL, United States; ^3^ Allergy and Immunology Care Center of South Florida, Miami Lakes, FL, United States; ^4^ Division of Allergy and Immunology, Nicklaus Children's Hospital, Miami, FL, United States; ^5^ Division of Pulmonary and Critical Care Medicine, Washington University School of Medicine, St. Louis, MO, United States; ^6^ University of North Carolina Eshelman School of Pharmacy, Chapel Hill, NC, United States; ^7^ Allergy & Asthma Network, Fairfax, VA, United States

**Keywords:** asthma, oral corticosteroids, survey, caregivers, safety, children, adrenal insufficiency, adverse events

## Abstract

**Introduction:**

Oral corticosteroids (OCS) are an essential component of asthma treatment, but their long-term effects can be serious and cumulative. Potentially serious effects include adrenal suppression and psychological effects. To mitigate side effects, OCS use should be minimized. A survey of caregivers in the US was conducted to determine their perceptions of OCS use for their child with asthma.

**Methods:**

Individuals who participated in US consumer research panels were invited to complete an online cross-sectional survey from October-November 2023. Eligible participants were caregivers of a child ages 6–17 years currently being treated for asthma by a healthcare provider and who had experienced an asthma attack, flare or exacerbation (AAFE) in the past 12 months.

**Results:**

Caregivers (*N* = 500) were racially and ethnically diverse (4% Asian, 18% Black, 63% White, 20% Hispanic/Latino). Responses indicated that 92% of caregivers' children had uncontrolled asthma. In the past 12 months, children were treated an average of 3.6 times for AAFE and received at least 3 OCS prescriptions. Overall, 61% of caregivers believed their child would be sick longer when having an AAFE if not treated with an OCS; 92% believed their child's asthma symptoms improved at least a moderate amount with OCS. In all, 69% and 54% reported familiarity with short-term and long-term side effects of OCS, respectively. Caregivers perceived OCS as relatively safe for their child. The most common short-term OCS side effect experienced by their child was mood changes. Caregivers were most concerned about mood changes and cardiovascular disease as short-term and long-term effects of OCS, respectively.

**Conclusions:**

OCS therapy is often used for AAFE and is perceived by caregivers as effective and safe. Caregivers and healthcare providers should be educated about side effects and the importance of optimizing asthma treatment to reduce OCS use.

## 1 Introduction

Asthma currently affects 7.5% of children ages 5–11 years and 8.7% of adolescents ages 12–17 years in the US (1). Inhaled corticosteroids (ICS) are the standard of care treatment for asthma, but uncontrolled disease and exacerbations still occur, usually in association with viral respiratory tract infections or allergen exposure (2, 3). Acute care is often sought to treat an exacerbation, and an average of 77 emergency department visits per 10,000 children and adolescents every year are due to asthma (4). During an exacerbation, oral corticosteroids (OCS) can be used to reduce airway inflammation. Treatment guidelines recommend a short course (3–5 days for children) of OCS for those who fail to respond to an increase in reliever or ICS dose, or who deteriorate rapidly, who have severely reduced lung function, or who have worsening asthma and a history of sudden severe exacerbations (2). OCS as a controller therapy should only be used as a last resort (2).

**Figure 1:**
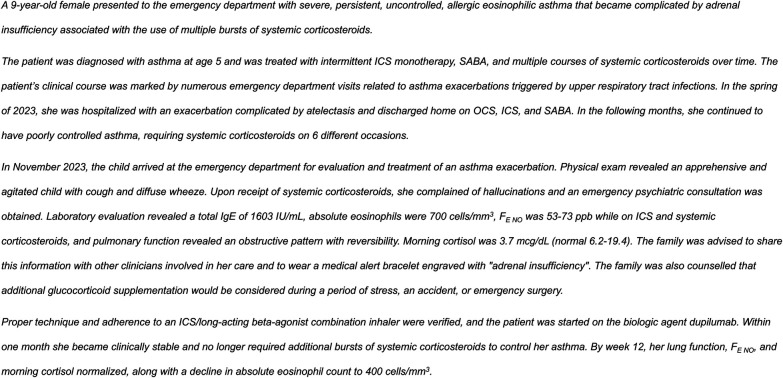
Case of OCS-associated adrenal insufficiency. F_E NO_; fraction of exhaled nitric oxide; ICS, inhaled corticosteroids; OCS, oral corticosteroids; SABA, short-acting beta-agonists.

Oral corticosteroids are an essential component of asthma treatment, but in addition to the increased risk of growth impairment in children (5), their long-term effects can be potentially serious and cumulative. Osteoporosis, cataracts, glaucoma, cardiovascular disorders, and Type 2 diabetes are just some of the physical comorbidities associated with long-term OCS use (6, 7). Furthermore, prolonged OCS use can lead to CD4T-cell lymphopenia and hypogammaglobinemia, placing the patient at risk of opportunistic infections and potentially a decreased humoral response to some vaccines (i.e., COVID-19 mRNA-based vaccines) (8, 9). OCS-associated adrenal suppression is another serious side effect that can result in adrenal insufficiency (Figure 1) (10). Psychological effects, such as psychosis, depression, anxiety, and manic episodes, are often overlooked but are common with OCS use in adults (6). Moreover, frequent short courses of OCS, not just long-term OCS use, are associated with complications in adults, adolescents, and children (11, 12). In a study of adults, the equivalent of as few as 4 lifetime short-term courses of OCS at a dose typically used to treat an asthma exacerbation can increase the risk of serious comorbidities (13). A study of almost 187,000 children and adolescents with asthma in the US found that 24% had received at least one OCS prescription in a 1-year period (14). Thus, OCS use should be minimized by optimizing ICS-based therapy (with or without a long-acting beta-agonist), ensuring proper ICS inhaler technique and good adherence to standard therapy, addressing modifiable exacerbation risk factors, and potentially using a biologic therapy (2).

Caregivers can be the best source of information regarding their child's medical history and repeated OCS use that is indicative of poorly controlled or hard-to-treat asthma. This is particularly true when the child is seen by multiple healthcare facilities (i.e., primary care, urgent care, emergency department) where the frequency of OCS prescriptions may be unknown among the various providers. In such cases, caregivers may need to advocate for their child to receive interventions that can reduce OCS use. However, if caregivers are not aware of the potential dangers of frequent or long-term OCS use, they will not know that changes in management are needed. A survey of caregivers in the US was conducted to better understand their knowledge of OCS and perceptions of OCS use for their child with asthma.

## 2 Methods

### 2.1 Survey participants

Individuals who had agreed to participate in US consumer research panels hosted by the market research firm, Dynata, were invited to complete an online cross-sectional survey from October to November 2023. Research panel participants from the US were invited by an email from Dynata that contained a link to the survey. Eligible participants from the panel were caregivers of a child ages 6–17 years currently being treated for asthma by a healthcare provider and who had experienced an asthma attack, flare or exacerbation (AAFE) in the past 12 months. Caregivers were required to be at least 18 years of age or have reached their state's age of consent (age 19+ years in AL and NE and age 21+ years in MS and PA). Each participant's identity was kept anonymous to ensure privacy, and each participant was assigned a unique identification number to prevent multiple survey completions.

### 2.2 Survey

Development of the survey questions was guided by information in the published literature. The questions were reviewed by a panel of allergy, immunology, pulmonology, and social sciences experts. Content and face validity were conducted through a group brainstorming process. A pretest of the final survey was conducted within the Dynata programming and management team and within Allergy & Asthma Network. A pilot launch of the survey was conducted in approximately 10% of the target participants before full launch of the survey. Dynata confirmed survey reliability through a series of quality checks for random responding and illogical or inconsistent responding. Survey reporting followed the Consensus-Based Checklist for Reporting of Survey Studies (15).

An institutional review board exemption was obtained from the Advarra Center for IRB Intelligence. Each participant consented that they had read and understood the disclosure statement and agreed to the requirements to participate in the survey. Dynata panel points, which can be exchanged for goods and services, were given as an incentive to survey participants.

The survey consisted of 40 questions pertaining to caregiver demographics, the child's asthma history and OCS use, and caregiver perceptions of OCS efficacy and safety (Supplementary Table S1). Questions were presented in the form of multiple choice, yes/no, or numbered scales.

Caregivers were given definitions of an asthma action plan (AAP), OCS, and short- and long-term side effects within the survey. Whenever these terms appeared in the survey, a corresponding definition appeared in a pop-up. The survey also provided relevant images of an AAP and different types of OCS packaging. The following definitions were given within the survey:

“An Asthma Action Plan is a written document or sheet of paper that provides guidance on how to help keep asthma symptoms in check and what to do if they get out of control. These written plans include a list of allergic triggers or irritants to avoid, how to know you are having an asthma attack and what to do next, which medicine to take and when, when to call your health care provider or the ambulance, and whom to contact in an emergency.”

“Oral corticosteroids (OCS), often referred to as oral steroids, are medicines that treat severe or uncontrolled asthma. They are available in pill, tablets that dissolve under the tongue or liquid form. Oral corticosteroids are not the same as anabolic steroids used in bodybuilding, nor are they the same as inhaled corticosteroids (ICS). Oral corticosteroids reduce inflammation quickly.”

“Short-term side effects are unwanted symptoms or health conditions that are a result of taking a medication. For example, experiencing mood changes while taking oral corticosteroids for an asthma flare.”

“Long-term side effects are unwanted symptoms or health conditions that are the result of taking a medication repeatedly for many years. For example, developing cataracts after taking oral corticosteroids for asthma flares. Long-term use of oral corticosteroids would be defined as taking more than 4 doses (courses) over a lifetime.”

### 2.3 Analyses

The goal of the survey was 500 participants. To obtain a diverse participant population, participant quotas for the survey were set at 39% aged 18–29 years, 31% aged 30–39 years, 17% aged 40–49 years, 13% aged 50+ years, 73% female, <1% American Indian or Alaska Native, 6% Asian, 18% Black, 12% Other race, 63% White, and 20% Hispanic/Latino of any race.

Children were designated as having uncontrolled asthma if their caregiver responded to survey questions that the child used a rescue inhaler ≥2 times per week, had nocturnal awakenings for asthma ≥2 times per month, or refilled their rescue inhaler ≥2 times a year (16). “Rescue inhaler” in this context referred only to a SABA and did not refer to an ICS plus SABA combination inhaler.

Analyses were primarily descriptive using frequencies and percentages. Responses were compiled for overall completers and by subgroups that contained >30 participants [i.e., income, race (e.g., non-Hispanic/Latino Black or non-Hispanic/Latino White), ethnicity (i.e., Hispanic/Latino of any race), child age, child missed school/not missed school, asthma controlled/uncontrolled]. Although the survey was not designed to evaluate differences between subgroups, chi-square analysis was used to determine statistical significance for between-group responses that appeared numerically different. Only responses from completed surveys were analyzed, therefore, no adjustment for non-response was necessary.

## 3 Results

### 3.1 Survey participants

A total of 3,084 individuals logged in to the survey, 690 passed the eligibility screening, and 500 caregivers completed the survey. The majority of caregivers were aged 18–39 years and female (Table 1). There was a diverse representation of races, and 20% of caregivers identified their ethnicity as Hispanic/Latino (Table 1). Over a third of participants lived in Southern US states and approximately half had a Bachelor's degree or higher (Table 1).

**Table 1:** Caregiver demographic characteristics.

Demographic, %	Total caregivers (*N* = 500)
Female	73
Age, years	
18–29	29
30–39	37
40–49	20
50+	14
Race	
American Indian/Alaska Native	1
Asian	4
Black	18
Multiracial	4
Native Hawaiian or Other Pacific Islander	5
Other	4
White	63
Hispanic/Latino	20
Education	
High school or less	14
Some college/Associate's degree	34
Bachelor's degree or higher	52
Household income, annual	
$0–$29k	16
$30k–$59k	22
$60k–$99k	44
$100k+	18
US geographic region	
Northeast	21
Midwest	21
South	37
West	21
Child's type of health insurance	
Group/private insurance	50
Medicaid	47
Other	1
None	2

### 3.2 Children's asthma history and medication use

Responses indicated that 92% of caregivers' children had uncontrolled asthma and 78% had missed school because of an asthma attack in the past 12 months. On a scale of 0–10, (0 = worst ever, 10 = best ever), caregivers rated their child's asthma most days of the week as an average of 6.3. Caregivers indicated their child had been treated for an AAFE an average of 3.6 times in the past 12 months and had received treatment in a doctor's office (64%), emergency department (36%), or urgent care facility (31%). Approximately two-thirds (64%) had an AAP for their child.

Only 5% of caregivers reported their child had not used an inhaled asthma controller medication in the past 12 months. Of those children who had seen a healthcare provider for an AAFE in the past 12 months (*n* = 445), the most common medication prescribed was an albuterol nebulizer (60%), followed by OCS liquid/syrup (33%), OCS pills (31%), OCS tablets that dissolve under the tongue (22%), budesonide nebulizer (22%), and corticosteroid injections (14%; Table 2). Based on the chi-squared test, there was a significant association between caregiver race/ethnicity and the prescription of OCS (*p* < 0.001). Children of non-Hispanic/Latino White caregivers were more likely than those of Hispanic/Latino caregivers to be prescribed OCS. The highest percentages receiving nebulizer prescriptions (both albuterol and budesonide preparations) were children of Hispanic/Latino caregivers or children of the lowest income level ($0–$29k; Table 2). Of those children who had received an OCS prescription, the average number of times it was prescribed was 3.3 for pills/tablets (*n* = 210) and 3.0 for liquid/syrup (*n* = 147). A significant association between caregiver race/ethnicity and the number of OCS pill/tablet prescriptions was observed (*p* < 0.05). The greatest average number of OCS pill/tablet prescriptions was in children of Hispanic/Latino caregivers (4.3 times; Table 3).

**Table 2:** Percentage of caregivers reporting medications prescribed for their child when seen by a healthcare provider for an AAFE in the past 12 months by subgroup.

Medication, % reporting	Total *n* = 445^a^	Annual Income				Ethnicity	Race		Child Age, y		Missed School		Asthma Control	
		$0–$29k *n* = 68	$30k–$59k *n* = 99	$60k–$99k *n* = 205	$100k+ *n* = 69	Hispanic/Latino *n* = 96	Non-Hispanic/Latino White *n* = 254	Non-Hispanic/Latino Black *n* = 74	6–11 *n* = 245	12–17 *n* = 200	Yes *n* = 369	No *n* = 76	Uncontrolled *n* = 424	Controlled *n* = 21
Albuterol nebulizer	60%	84%	74%	48%	54%	76%	50%	69%	56%	66%	60%	58%	59%	76%
OCS liquid/syrup	33%	40%	35%	31%	30%	22%	35%	41%	36%	30%	33%	33%	34%	24%
OCS pill	31%	35%	26%	29%	36%	22%	32%	34%	27%	35%	32%	26%	31%	24%
OCS tablet that dissolves under the tongue	22%	16%	15%	25%	32%	17%	26%	22%	24%	21%	22%	22%	22%	19%
Budesonide nebulizer	22%	27%	23%	21%	19%	30%	19%	20%	21%	24%	23%	16%	23%	10%
Corticosteroid injection	14%	10%	8%	17%	15%	17%	14%	8%	14%	13%	14%	12%	14%	0
Other	2%	4%	2%	1%	1%	2%	2%	3%	2%	2%	1%	7%	2%	10%

AAFE, asthma attack; flare, or exacerbation; OCS, oral corticosteroids.

^a^ Base number of caregivers reporting their child saw a healthcare provider for an AAFE in the past 12 months.

**Table 3:** Percentage of caregivers reporting how many times their child received an OCS pill or tablet prescriptions in the past 12 months by subgroup.

Number of OCS Prescriptions, % reporting	Total *n* = 210^a^	Annual Income				Ethnicity	Race		Child Age, y		Missed School		Asthma Control	
		$0–$29k *n* = 31	$30k–$59k *n* = 39	$60k–$99k *n* = 101	$100k+ *n* = 37	Hispanic/Latino *n* = 31	Non-Hispanic/Latino White *n* = 132	Non-Hispanic/Latino Black *n* = 38	6–11 *n* = 114	12–17 *n* = 96	Yes *n* = 177	No *n* = 33	Uncontrolled *n* = 202	Controlled *n* = 8
1	19%	26%	21%	14%	22%	10%	19%	26%	17%	22%	16%	36%	17%	62%
2	27%	38%	31%	23%	24%	23%	27%	21%	30%	23%	25%	33%	26%	38%
3	22%	10%	15%	31%	19%	23%	23%	18%	19%	26%	24%	15%	23%	0
4	15%	16%	8%	16%	19%	10%	15%	18%	19%	26%	17%	3%	15%	0
5–9	13%	10%	10%	14%	16%	31%	12%	1%	14%	11%	4%	3%	15%	0
10+	4%	0	15%	2%	0	3%	4%	5%	6%	2%	14%	10%	4%	0
AVERAGE	3.3	2.6	3.9	3.3	3.1	4.3	3.2	2.8	3.8	3.1	3.4	2.6	3.4	1.4

AAFE, asthma attack, flare, or exacerbation; OCS, oral corticosteroids.

^a^ Base number of caregivers reporting their child received an OCS pill or tablet prescription for an AAFE in the past 12 months.

### 3.3 Caregiver perceptions of OCS use and efficacy

Nearly all (93%) of the surveyed caregivers correctly believed that OCS is used to treat AAFEs. Overall, 61% of caregivers believed their child would be sick longer when having an AAFE if not treated with an OCS and 57% expected they would receive a prescription, including a prescription for OCS, for their child's AAFE when they visited a healthcare provider. A higher percentage of White caregivers had an expectation of an OCS prescription than Black and Hispanic/Latino caregivers (69% vs. 55% vs. 53%, respectively). A summary of open-ended responses explaining why an OCS prescription was expected is shown in Table 4. Over two-thirds of caregivers (68%) whose child was prescribed an OCS (*n* = 310) reported they filled it every time, and 69% of those who filled the prescription at least some of the time (*n* = 299) said their child finished the full prescribed regimen.

**Table 4:** Examples of reasons caregivers expect an OCS prescription for their child's AAFE based on open-ended responses.

Reasons caregivers expect an OCS prescription	
• Prevents long-term lung damage	• Allows the child to sleep better
• Lowers chance of another attack	• Always received in the past
• Controls inflammation	• Only medication that works
• Quick relief	• Albuterol doesn’t control the flare-ups
• Prevents hospitalization	• Available as pills, tablets, or syrup
• Makes life more predictable	• Previous flare-ups required hospitalization
• Stops attacks before they worsen	• Have helped me too

AAFE, asthma attack, flare, or exacerbation; OCS, oral corticosteroids.

When asked if their child's healthcare provider had prescribed too many OCS for their child's asthma treatment, 37% of caregivers agreed and 41% disagreed; 84% agreed that their child's healthcare provider was aware of the number of times OCS had been prescribed in the past 12 months.

The majority of caregivers (58%) whose children took at least a portion of their OCS prescription (*n* = 299) believed their child's asthma symptoms improved significantly and 34% believed symptoms improved a moderate amount.

### 3.4 Caregiver perceptions of OCS safety

**Figure 2:**
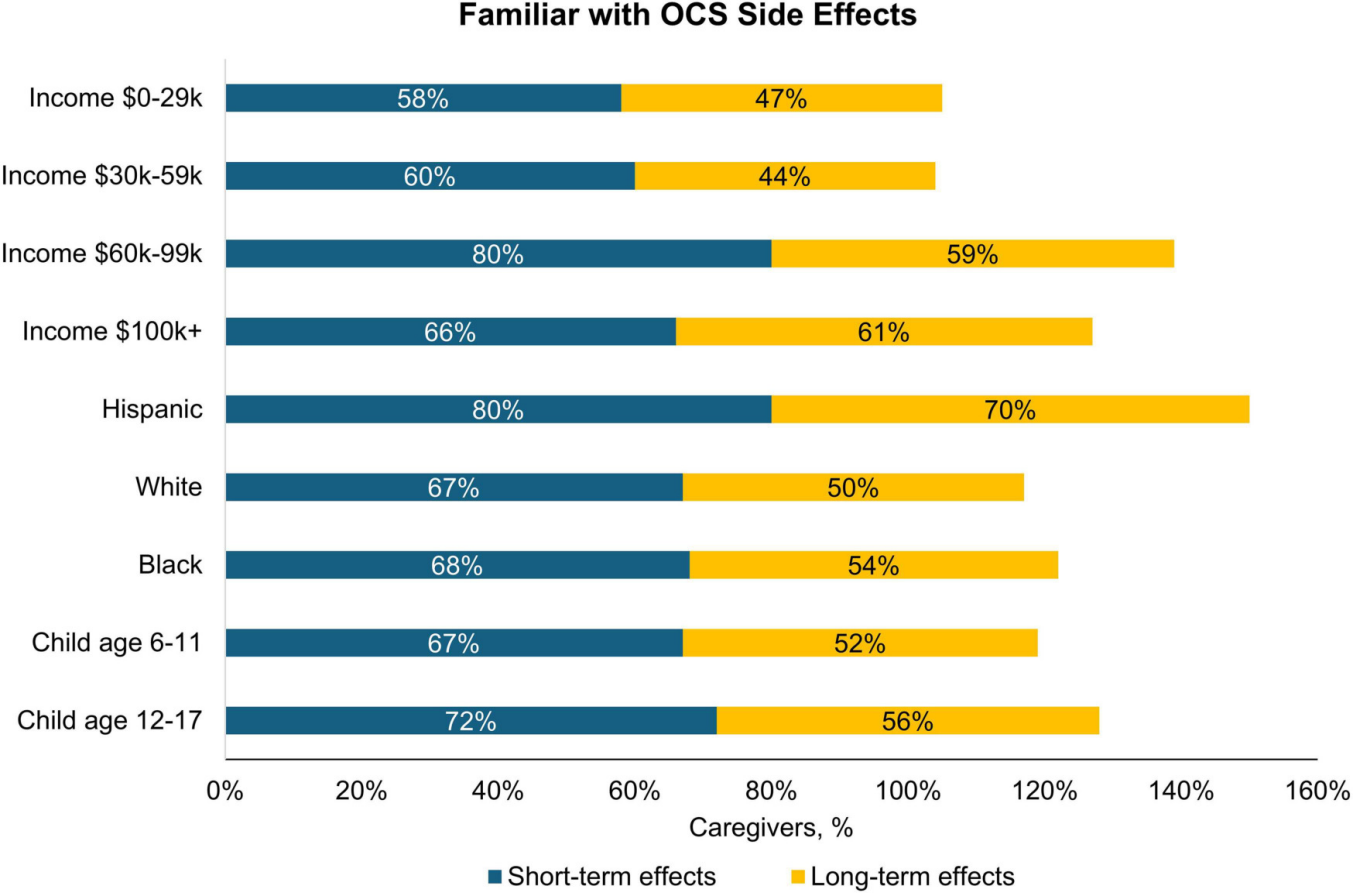
Percentage of caregivers who were familiar with short-term and long-term side effects of OCS by subgroup (*n* = 500). OCS, oral corticosteroids.

**Figure 3:**
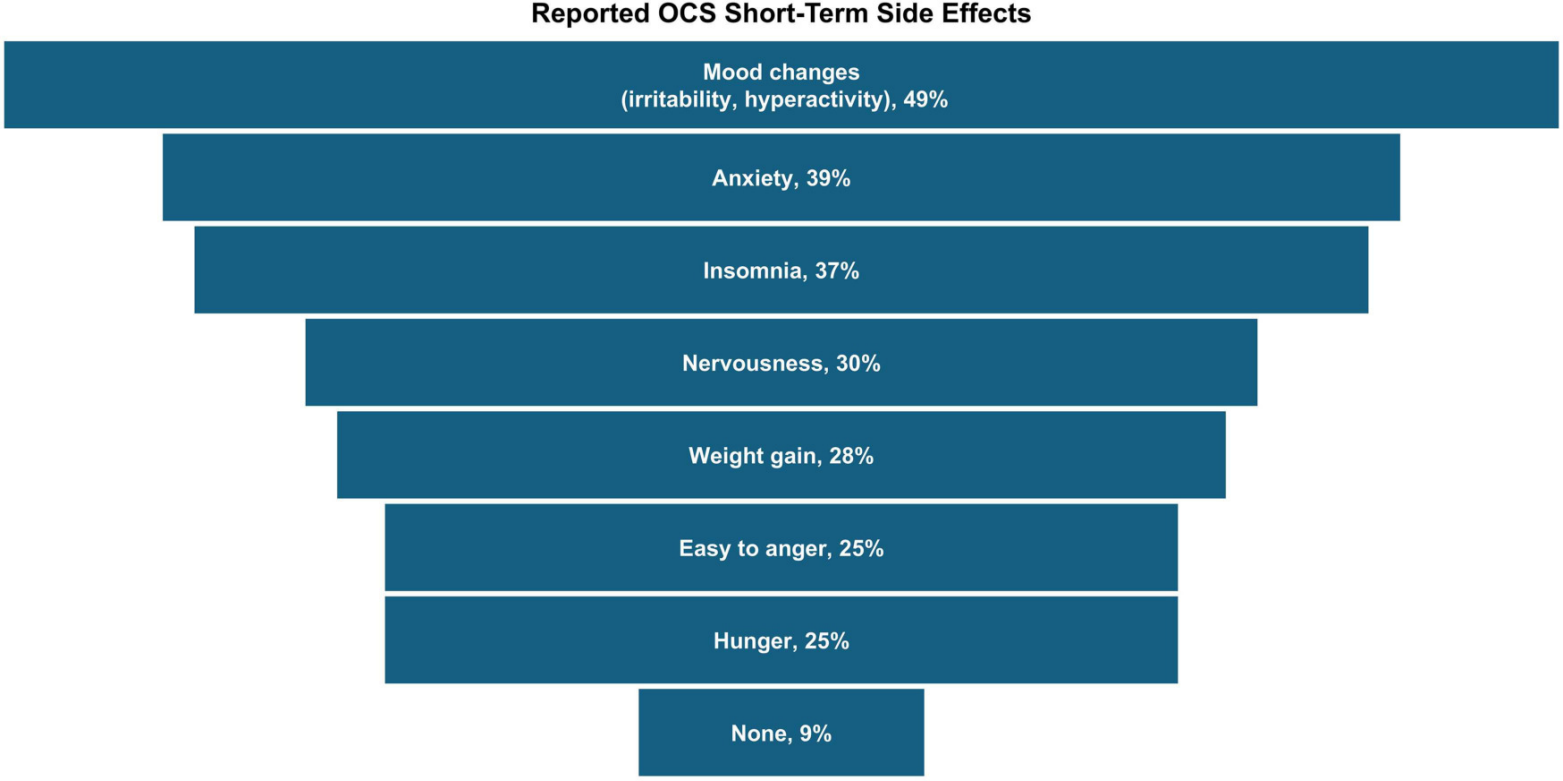
Short-term side effects experienced by children following OCS treatment (*n* = 233, child took OCS and caregiver familiar with short-term side effects). Caregivers could select multiple side effects. OCS, oral corticosteroids.

**Figure 4:**
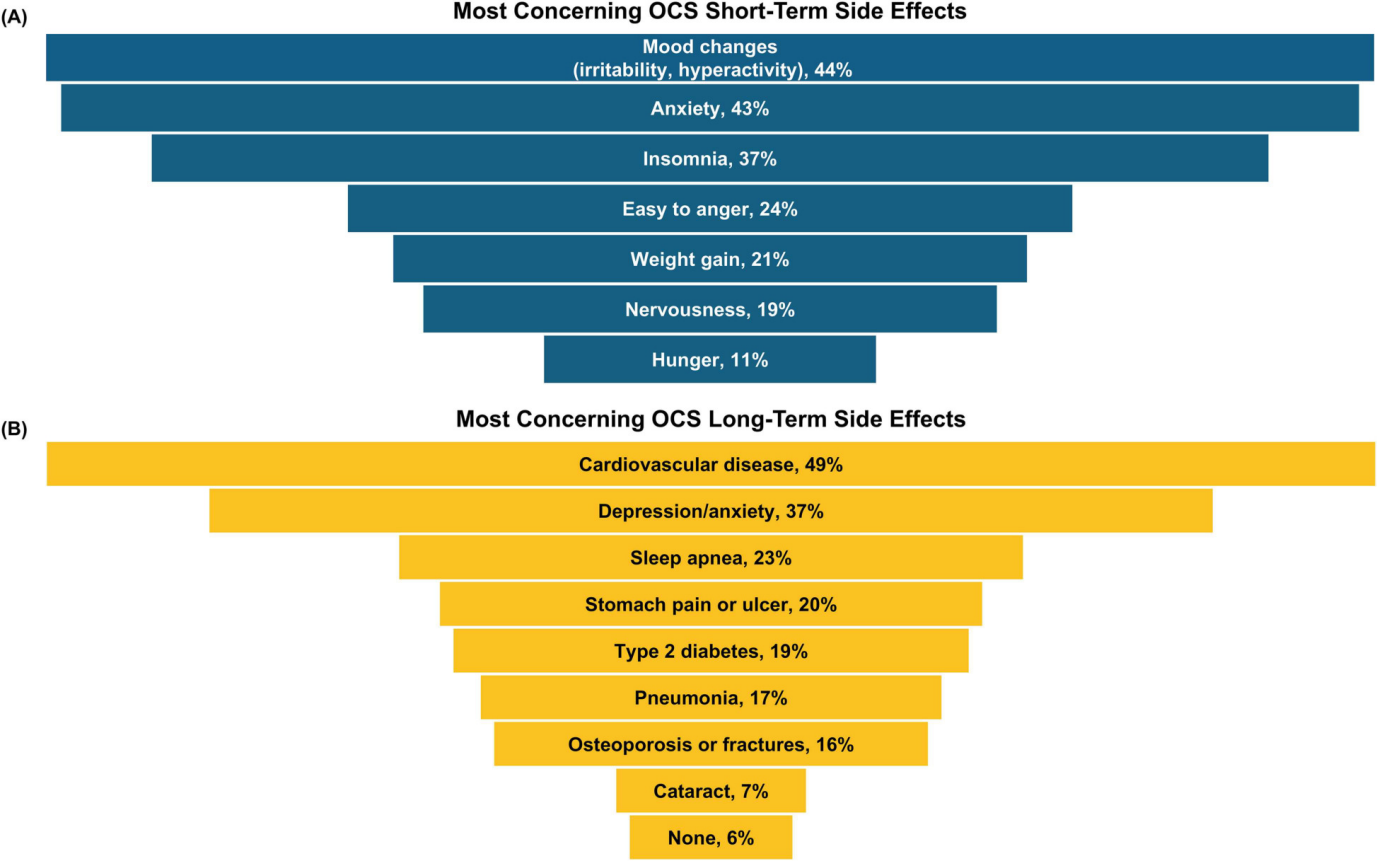
Top-ranked **(A)** short- and **(B)** long-term side effects of OCS that caregivers were most concerned about (*n* = 500 responses). Caregivers ranked their top 2 concerns from a provided list of side effects. OCS, oral corticosteroids.

About half (56%) of all caregivers agreed their healthcare provider had informed them about the cumulative long-term side effects of OCS; only 69% and 54% reported familiarity with short-term and long-term side effects of OCS, respectively. A higher percentage of Hispanic/Latino caregivers reported being familiar with side effects than Black or White caregivers (Figure 2). Caregivers perceived OCS as relatively safe for their child; on a scale of 0–10 (0 = harmful to my child's health, 10 = safe for treating my child's asthma), the average caregiver score was 7.2. Of the list of side effects provided in the survey questions, the most common short-term OCS side effect experienced by their child was mood changes (e.g., irritability, hyperactivity, 49%), followed by anxiety (39%) and insomnia (37%; Figure 3). The most concerning listed short-term OCS side effect to caregivers was mood changes (44%; Figure 4A). Hispanic caregivers were most concerned about insomnia (41%), Black caregivers about mood changes (55%), and White caregivers about anxiety (46%). The most concerning listed long-term OCS side effect to caregivers was cardiovascular disease (49%; Figure 4B). Cardiovascular disease was the most concerning long-term side effect for Black and White caregivers (51% and 49%, respectively), and for Hispanic/Latino caregivers was depression/anxiety (42%).

## 4 Discussion

Children of this racially-diverse caregiver population in the US had a high prevalence of uncontrolled asthma and received frequent OCS prescriptions. Caregivers believe that OCS is beneficial and safe in treating their child's asthma, despite a high percentage reporting side effects with their child's OCS use and a large proportion reporting unfamiliarity with known side effects.

The relatively high number of acute care visits and OCS prescriptions reported by the caregivers corroborates an uncontrolled population, despite the fact that nearly all the children were reported to be using a controller medication. Adherence to the controller medication was not assessed in the survey, and poor adherence could at least partially explain the high prevalence of uncontrolled disease (17, 18).

Caregivers believed that OCS improved their child's asthma symptoms and that their child would remain sick for an extended period of time if they did not receive an OCS. Thus, there was an expectation among over half of caregivers, and especially among White caregivers, that their child would receive an OCS for an AAFE. This parallels findings from a retrospective database analysis of adolescents and adults with asthma in the US, of whom 65% were prescribed an OCS during a 2-year follow-up period (19). In a large survey of adolescents and adults with asthma in the US, 35% reported they had taken OCS in the past year (20). These data together indicate that prescribing of an OCS is common practice. Therefore, caregiver's expectation of an OCS prescription is understandable, particularly because they observe improvements in their child's symptoms with OCS. Additionally, OCS is low-cost and easily accessible, which enhances its perceived benefit in the eyes of caregivers (21). This high parental expectation likely influences healthcare provider's prescription patterns, leading to frequent use and potential overuse of OCS.

Despite nearly half of caregivers reporting mood changes as a concerning side effect, some may perceive the rapid improvement in asthma symptoms following OCS use as indicative of safety, suggesting a potential disconnect between short-term relief and awareness of side effects. When rating the safety of OCS for their child, it is possible that the caregivers considered the short-term side effects listed in the survey, which were mainly psychological effects, as relatively benign compared with the listed potential long-term effects (e.g., cardiovascular disease). However, while caregivers may regard irritability, hyperactivity, anxiety, and insomnia as less severe than physical side effects, these psychological effects can substantially diminish the quality of life for children and adolescents with asthma. A study of patients with asthma found that insomnia and mood changes were among the top side effects of OCS they most wished to eliminate (22).

Non-psychological complications that may be perceived by caregivers as more serious are also associated with short-term OCS use. A retrospective study of adults in the US found a significant increase in the rates of sepsis, venous thromboembolism, and fracture within 30 days of starting OCS (23). A similar study of children in Taiwan found that the risk of gastrointestinal bleeding, sepsis, and pneumonia was increased 1.4–2.2-fold within 5–30 days of beginning a single short-course of OCS (24). Hypothalamic pituitary adrenal axis suppression and adrenal insufficiency are other known side effects associated with systemic corticosteroids. Adding to this burden, patients are commonly prescribed inhaled, intranasal, and topical corticosteroids to address coexisting atopic conditions (AKA “steroid stacking”), increasing the overall systemic exposure and the potential risk of adverse events (21). A morning cortisol level can serve as a valuable screening method for adrenal insufficiency, and an evaluation with an endocrinologist may be useful for patients suspected to have adrenal insufficiency.

A large proportion of the surveyed caregivers were not familiar with the side effects of OCS, despite multiple OCS prescriptions for their child. Medication side effects should be part of shared decision-making conversations between caregivers and healthcare providers. Armed with this information, caregivers can be vigilant for the signs and symptoms of serious OCS-related complications. In addition, caregivers may be more diligent in monitoring adherence to controller medication if they understand that nonadherence places their child at greater risk of OCS exposure and its associated complications.

In recognition of the burdens and harms associated with OCS use, there are a number of organizations that have called for the adoption of steroid-sparing and steroid stewardship strategies (21, 25–29). There are steroid-sparing biologic therapies for asthma that are now approved for children ages 6 years and up. Assuming issues of adherence, inhaler technique, and modifiable risk factors have already been addressed, following treatment guidelines for step-up therapy to improve asthma control can reduce the need for OCS (21, 30, 31). Caregivers and healthcare providers should be educated about the importance of optimizing asthma therapy to reduce exposure to systemic corticosteroids and both short- and long-term adverse effects. Reducing the need for short-term courses or maintenance OCS should be a key goal in the management of any patient with asthma to mitigate the effects of cumulative corticosteroid exposure.

A limitation of the survey is the risk of bias since participants were part of a consumer research panel and had access to the internet. Thus, results may not be generalizable to the general population of asthma caregivers in the US. The results of this survey may also not be generalizable to caregivers of children without frequent AAFEs. Caregivers who are more aware of the side effects of OCS may be more diligent to ensure medication adherence and advocate for interventions that lead to better asthma control and reduce or avoid OCS use. Another limitation of the survey was that side effects such as growth suppression, adrenal insufficiency, or acne that are more relevant for children and adolescents were not evaluated.

## 5 Conclusions

OCS therapy is often used for an AAFE and is perceived by caregivers as effective and safe. The perception of safety contradicts the high percentage of caregivers reporting that their child experienced mood changes and anxiety with OCS use and caregiver concerns about cardiovascular effects with long-term use. Caregivers and healthcare providers should be educated about side effects and the importance of optimizing asthma treatment to reduce the use of OCS.

## Data Availability

The original contributions presented in the study are included in the article/Supplementary Material, further inquiries can be directed to the corresponding author.
