# Supplementary material for: Perceptions of oral corticosteroid use for children with asthma in a survey of US caregivers
Source: Front Pediatr. 2025 Oct 7;13:1608425. doi: 10.3389/fped.2025.1608425 (PMC12537427; doi:10.3389/fped.2025.1608425)
Supplement: Supplementary file 1 [file Supplementaryfile1.pdf]

Supplementary Material

1 Supplementary Methods.

Table S1. Survey questions.

1. What is your age? (Please enter a whole number)

1	17 or younger
2	18 - 29
3	30 - 39
4	40 - 49
5	51 - 64
6	65 - 70
7	71 or older

2. In what state is your primary residence? (Select one) **DROP DOWN LIST FOR US STATES AND DC**

3. With which of the following do you most identify?

1	Male
2	Female
3	Non-binary

4	Other
5	Prefer not to answer

4. Do you consider yourself to be of Hispanic or Latino origin/culture?

1	Yes
2	No

5. Which of the following best describes your racial identity? (Select one)

1	White/Caucasian
2	Black/African-American
3	Asian/Asian-American
4	American Indian or Alaska native
5	Native Hawaiian or Other Pacific Islander
6	Multiracial
7	Other (Please specify)

6. What is the highest level of education that you have had the opportunity to complete?

1	Less than high school degree
2	High school degree or equivalent (e.g., GED)

3	Some college but no degree
4	Associate degree
5	Bachelor’s degree
6	Graduate, law, or doctorate degree
7	Prefer not to answer

7. Approximately what was your annual gross household income last year before taxes (2022)?

1	$0 – $9,999
2	$10,000 – $19,999
3	$20,000 – $29,999
4	$30,000 – $39,999
5	$40,000 – $49,999
6	$50,000 – $59,999
7	$60,000 – $69,999
8	$70,000 – $79,999
9	$80,000 – $89,999
10	$90,000 – $99,999
11	$100,000 or more
12	Prefer not to answer

8. Are you the parent or caregiver of a child under age 18?

1	Yes
2	No

9. For which of the following conditions, if any, are any of your children currently being treated by a healthcare provider? (Select all that apply)

1	Asthma
2	Allergies
3	COVID-19
4	Eczema / Atopic Dermatitis
5	Diabetes
6	AIDS
7	Mononucleosis
8	Cancer
9	Other (Please specify)
10	None of the above

10. How old are your children who are being treated by a healthcare provider for the following conditions? (Select one or more age categories for each of the following)

		Age 0-5	Age 6-11	Age 12-17

1	Asthma			
2	Allergies			
3	COVID-19			
4	Eczema / Atopic Dermatitis			
5	Diabetes			
6	AIDS			
7	Mononucleosis			
8	Cancer			

For the rest of this survey, please answer regarding your child (or just one of your children) who is age 6-11 or age 12-17 and has asthma.

11. An asthma attack, exacerbation or flare occurs when signs and symptoms associated with asthma happen. When, if at all, was the last time that your child with asthma experienced an asthma attack, exacerbation, or flare? (Select one)

1	Within the past 6 months
2	In the past 7-12 months
3	About 13-24 months ago (1 to less than 2 years ago)
4	About 25-36 months ago (2 to less than 3 years ago)
5	3 years ago or more
6	My child has never experienced an asthma attack, exacerbation or flare

12. Which of the following types of healthcare providers(s) has your child seen for asthma care in the last 12 months? (Select all that apply)

1	Allergist
2	Family Practice or Primary Care Physician
3	Pediatrician
4	Respiratory Therapist
5	Pulmonologist
6	Nurse Practitioner
7	Physician Assistant
8	Registered Nurse
9	Other (Please specify)
10	My child has not seen a healthcare provider for asthma in the past 12 months

END OF SCREENER

13. When you think about your child's asthma most days of the week, how would you rate your child's asthma on the following scale?

Worst<br>asthma		Best<br>asthma

ever										ever
0	1	2	3	4	5	6	7	8	9	10

14. Please answer "yes" or "no" to the following questions.

		Yes 1	No 2
A	Does your child use their reliever/rescue inhaler more than twice a week for asthma symptoms?		
B	Does your child wake up at night because of asthma symptoms more than two times a month?		
C	Does your child refill their quick-relief inhaler more than two times a year?		

15. Has your child missed a school day (or usual daily activities) because of an asthma attack/flare in the past 12 months?

1	Yes
2	No

16. Does your child have an asthma action plan?

1	Yes

2	No

17. Please indicate your strength of agreement with the following statements.

		Strongly Disagree 1 2 3 4 Strongly Agree 5
A	I am comfortable using my child's Asthma Action Plan.	
B	I would like more information about how to use the Asthma Action Plan for my child.	

18. Which of the following conditions do you believe oral corticosteroids are used to treat? (Select all that apply)

1	Asthma flares
2	Eczema flares
3	Common cold, flu
4	Ear or sinus infection
5	Ankle or wrist sprain
6	Other (Please specify)
7	None of these
8	I don't know

19. How strongly do you believe that oral corticosteroids, when prescribed by a healthcare provider for asthma, are either harmful or safe for your child? Select one response on the

scale below indicating which statement you agree with the most; the closer your selection is to the endpoint, the stronger your belief.

Harmful to my child's health						Safe for treating my child's asthma				
0	1	2	3	4	5	6	7	8	9	10

20. Which of the following asthma (inhaled) medication regimens has your child with asthma used in the past 12 months? (Select all that apply)

1	Advair Diskus (fluticasone priopionate and salmeterol)
2	Advair HFA (fluticasone priopionate and salmeterol)
3	AirDuo Digihaler (fluticasone propionate and salmeterol)
4	AirDuo Respiclick (fluticasone propionate and salmeterol)
5	Alvesco HFA (ciclesonide)
6	Armonair Digihaler (fluticasone propionate)
7	Arnuity Ellipta (fluticasone furoate)
8	Asmanex HFA (mometason furoate)
9	Asmanex Twisthaler (moemtasone furoate)
10	Breo Ellipta (flutcasone furoate and vilanterol)

11	Dulera (mometasone furoate and formoterol fumarate)
12	Flovent Diskus (fluticasone)
13	Flovent HFA (fluticasone)
14	Pulmicort Flexhaler (budesonide)
15	QVAR RediHaler (beclomethasone dipropionate HFA)
16	Serevent Diskus
17	Spiriva Respimat (tiotropium)
18	Symbicort (budesonide and formoterol)
19	Wexala Inhub (fluticasone propionate and salmeterol)
20	Other (Please specify)
21	None of these

21. How strongly do you agree with the following statements?

		Strongly Disagree 1 2 3 4 5 Strongly Agree
A	I believe that my child with asthma will be sick longer when having an asthma flare if not treated with an oral corticosteroid.	

B	When I visit a healthcare provider (e.g., doctor, nurse practitioner, physician assistant) for my child's asthma flare, I expect a prescription for medication, including a prescription for an oral corticosteroid.	

22. Please explain why you expect a prescription for an oral corticosteroid for your child when visiting a healthcare provider for an asthma flare. **OPEN END TEXT BOX**

23. When your child experienced an asthma flare in the past 12 months, did you see a healthcare provider (a doctor, nurse practitioner or physician assistant) in any of the following locations? (Select all that apply)

1	Doctor's office or clinic
2	Urgent care facility
3	Hospital emergency room
4	I did not take my child to see a healthcare provider; we treated the flare at home

24. Approximately how many times in the past 12 months has your child seen a healthcare provider (in a doctor's office, urgent care facility or hospital emergency room) for an asthma flare? (Please enter a whole number)

25. When your child saw a healthcare provider for an asthma flare in the past 12 months, which of the following medications were prescribed? (Select all that apply)

1	Oral corticosteroid pill (prednisone or Medrol)

2	Oral corticosteroid tablets that dissolve under the tongue (prednisone or Medrol)
3	Oral corticosteroid liquid/syrup (prednisone, prednisolone, dexamethasone)
4	Corticosteroid injection
5	Albuterol nebulizer
6	Budesonide nebulizer
7	Other (Please specify)

26. A. Approximately how many times in the past 12 months was your child prescribed an oral corticosteroid (pills or dissolving tablet)? (Please enter a whole number)

    B. Approximately how many times in the past 12 months was your child prescribed an oral corticosteroid (liquid or syrup)? (Please enter a whole number)

27. When your child was prescribed oral corticosteroids, was the prescription filled?

1	Yes, every time
2	Yes, most of the time
3	Yes, some of the time
4	No

28. When using prescribed oral corticosteroids, did your child finish all the medicine, or if your child is currently taking this medicine, do you expect to finish all the medicine? (Select one)

1	My child finished / will finish all the medicine
2	My child finished / will finish most of the medicine
3	My child finished / will finish a little of the medicine
4	My child did not / will not take any of the medicine

29. When your child was taking oral corticosteroids over the past 12 months, what was the outcome? (Select one)

1	My child's asthma symptoms improved significantly
2	My child's asthma symptoms improved a moderate amount
3	My child's asthma symptoms improved only slightly
4	My child's asthma symptoms did not improve at all
5	My child's asthma got worse

30. Are you familiar with the short-term side-effects and long-term side effects of oral corticosteroid use?

		Yes, I am familiar	No, I am not familiar	I'm not sure
A	Short-term side effects			
B	Long-term side-effects			

31. Over the past 12 months, which of the following oral corticosteroid <u>short-term</u> side effects has your child with asthma experienced? (Select all that apply)

1	Insomnia – inability to go to sleep.
2	Hunger
3	Anxiety
4	Nervousness
5	Easy to anger
6	Weight gain
7	Mood changes (irritability, hyperactivity)
8	Other (Please specify)
9	None

32. Of the following <u>short-term</u> side effects of oral corticosteroid use, which two are you most concerned about? (Please select and rank order your top two; rank #1 is the most concerning to you)

1	Insomnia – inability to go to sleep.
2	Hunger
3	Anxiety
4	Nervousness

5	Easy to anger
6	Weight gain
7	Mood changes (irritability, hyperactivity)
8	Other (Please specify)

33. Of the following long-term side effects of oral corticosteroid use, which two are you most concerned about? (Please select and rank order your top two; rank #1 is the most concerning to you)

1	Cardiovascular disease (e.g., heart failure, myocardial infarction, cerebral vascular accident, coronary vascular disease)
2	Osteoporosis or fractures
3	Pneumonia
4	Depression/ anxiety
5	Sleep apnea
6	Cataract
7	Type 2 Diabetes
8	Stomach pain or ulcer
9	Other (Please specify)
10	None

34. How strongly do you agree with the following?

		Strongly Disagree 1 2 3 4 5 Strongly Agree
A	My child's asthma healthcare provider (e.g., doctor, physician assistant, nurse practitioner) has prescribed too many oral corticosteroids to my child for asthma treatment.	
B	My child's asthma healthcare provider (e.g., doctor, physician assistant, nurse practitioner, etc.) is aware of the number of times my child has been prescribed oral corticosteroids in the last 12 months.	
C	My child's asthma healthcare provider is aware of my child's UC and ER visits that include prescribed oral corticosteroids.	
D	My child's asthma healthcare provider (e.g., doctor, physician assistant, nurse practitioner, etc.) has informed me about the cumulative long-term side effects of oral corticosteroid use.	

35. Please explain why you feel that your child's healthcare provider has prescribed too many oral corticosteroids to treat your child's asthma.

36. Please provide any final comments you may have regarding anything else would you want us to know about your use of OCS for your child. (A response to this question is not required)

37. Where would you go to learn more about asthma, asthma medication or asthma treatment (Select all that apply)

1	Online searches
2	Word of mouth; friends and family
3	Brochures
4	Social media sites (Twitter, Facebook, Instagram, etc.)
5	Medical websites (WebMD, etc.)
6	My Allergist's practice website
7	Professional medical associations (AMA, etc.)
8	Patient organizations (Allergy and Asthma Network, etc.)
9	My doctor or healthcare provider
10	My family/friends
11	My pastor or other trusted advisor
12	Other (Please specify)
13	I am not interested in learning more

38. Which of the following best describes the neighborhood where your primary residence is located? (Select one)

1	Major metropolitan area
2	Small city
3	Suburb
4	Rural area

39. What is the 5-digit zip code of your primary residence?

40. Which of the following best describes the type of health insurance coverage that your child currently has. (Select one)

1	Group/private insurance obtained by me personally or through an employer or COBRA
2	Medicaid
3	Other (Please specify)
4	I don’t have any health insurance coverage
5	I don’t know
